# A Novel Approach for Comparison of Reception Performance in a Technique Test and Small-Sided Games

**DOI:** 10.3390/sports9050066

**Published:** 2021-05-17

**Authors:** Arne Sørensen, Vidar Sørensen, Terje Dalen

**Affiliations:** 1 Department of Physical Education and Sport Science, Nord University, 7600 Levanger, Norway; terje.dalen@nord.no; 2 Malvik High School, 7560 Vikhammer, Norway; vidso@trondelagfylke.no

**Keywords:** soccer, skills, reception, technical-tactical assessment, SSG

## Abstract

The purpose of this study was to evaluate the correlation between soccer players’ performance of receptions of passes in tests of both isolated technical skills and more match-realistic situations in small-sided games (SSGs). In addition, this study investigated whether the involvement in SSGs (number of receptions) correlated with the quality of receptions in the respective SSGs. The participants were 13 male outfield youth soccer players from teams in the first division of the regional U18 league. The quality of receptions was scored by educated coaches according to set criteria of performance. Statistical analyses of correlations were determined using Spearman’s rank-order correlation coefficient (r_s_). The main results were (1) a significant correlation in the quality of ball reception between 4vs1 SSGs and 5vs5 SSGs (r_s_ = −0.61, *p* < 0.01) and (2) a trend towards moderate correlation between the quality of ball reception using a ball projection machine and 5vs5 SSGs (r_s_ = −0.48, *p* = 0.10). (3) A significant correlation was found between the number of receptions in 5vs5 SSGs and the quality score of receptions in 5vs5 SSGs (r_s_ = −0.70, *p* < 0.01). The trend towards moderate correlations between 5vs5 SSGs and the isolated technical reception test could imply the importance of training in the technical aspects of ball reception. Moreover, it seems as though the players with the best reception performance are the players who are most involved in SSGs, that is, having the most receptions.

## 1. Introduction

To have any success in soccer, players are dependent on a high level of ability based on such factors as physical, mental, technical, and tactical skills [1], where skill is considered to have the largest contribution to match performance [2,3]. Reliable and valid methods for measuring skills in soccer are essential to identify talented players and to evaluate potential effects of training. Accordingly, the testing of isolated technique skills has been criticised [2,4]. These authors claim that isolated technique tests have low ecological validity and provide coaches and researchers only with information about isolated player techniques rather than the skills necessary to perform in real-life situations. During soccer matches, players make continuous decisions about what to do in different situations depending on their perceptions and the actions of teammates and opponents [5]. In an attempt to ensure high test reliability, standardised tests of passing, dribbling, and shooting have been used to investigate the technical skills of soccer players, and these have been proven to be good strategies with which to differentiate between players at different levels [6,7,8,9]. Investigations have found the Loughborough Soccer Passing Test (LSPT) to have acceptable test–retest reliability and the ability to categorise players into different performance levels [10]. However, a high score on the LSPT had low validity as to passing skills in matches with male players in a U18 team [11]. Serpiello et al. (2027) investigated overall success rates for all passes during matches compared to performance on the LSPT, with the passing skills scores based on successful vs. unsuccessful passes.

In order to investigate the validity of technique tests, researchers used four isolated tests—(1) dribbling with pass, (2) dribbling speed, (3) passing, and (4) shooting accuracy—to assess the performance in 5vs5 SSG of elite youth male players [12]. The scores were evaluated by educated soccer coaches, and only the dribble-with-pass technique test (i.e., test 1) correlated with the performance in 5vs5 SSG.

Some studies have found that high performance in technical skills can predict performance in SSG or in full-scale soccer matches [13,14,15,16,17]. For example, dribbling speed along curved paths was shown to predict a higher possibility of scoring a goal in a one-on-one soccer match [14,17]. Moreover, high validity of performance ratings in technical skills compared to performance in SSG was also reported in a study of young players (7-to-13 years old) [15]. These results showed that technical skills could explain 60% of the average number of successful passes and percentage passing success [15]. In addition, performance in one-on-one soccer-tennis matches was compared to performance in five 11-on-11 soccer matches, and the results showed that players with high levels of success in soccer-tennis were likely to perform better in soccer matches [16]. In addition to these findings, there are several other factors that play decisive roles in player performance during matches, e.g., the player’s tactical level [18], the quality of teammates and opponents [19], and fatigue [8,20,21].

Even high-performing players show variability in their soccer performance [21]. Liu et al. (2016) found that players for weak teams in the Spanish first division showed a high variation in most of the attack-related match actions and a lower variation in the defence-related match actions and events, including interception, clearance, and yellow cards [19].

In top international soccer matches, commentators often use the expression, “first touch let him down”. This phrase emphasises that the quality of the first touch/reception is decisive for the player’s next action, which is often to pass [22]. The reason for ball interception is often that the reception is in the wrong direction according to good tactics or that the reception is too far from the player [2]. There is a lack of scientific studies evaluating the isolated technique of reception, probably due to methodological challenges in making accurate measurements. Top players execute, on average, 60 touches of the ball during a match [21]. Therefore, good quality of receptions is crucial for controlling and keeping possession of the ball, challenging an opponent, and making a shot at a goal. Good ball reception was found in a notational analysis of performance to be, after dribbling, the most important skill in creating goal-scoring opportunities in female college soccer [22]. The quality of ball reception in soccer has been investigated in two studies in which educated soccer coaches evaluated the quality of receptions, giving them scores in SSG from 1 to 5 for each reception [12,23]. The advantage of this type of evaluation is that player skills in SSG are measured in match-realistic environments and include both technical and tactical factors as part of the test score.

Reliable and valid methods for measuring skills in soccer are essential to identify talented players and to evaluate potential effects of training. Still, the main problem related to testing technical skills in soccer is that players’ decision-making is limited or absent. Few studies exist of evaluating performance in receptions in different soccer activities. Therefore, the main aim of this study was to explore the correlation between soccer players’ performance of receptions in tests both as an isolated technical skill and in more match-realistic situations in 4vs1 SSGs and 5vs5 SSGs. The use of SSGs in practice for soccer teams is popular both for elite players and in youth soccer [24].

In addition, this study explored the validity of quality of ball reception in 4vs1 SSG in relation to quality of ball reception in 5vs5 SSG, and it investigated whether the involvement in SSGs (number of receptions) correlated with the quality of receptions in the respective SSGs.

## 2. Materials and Methods

### 2.1. Participants

This experiment collected data from 13 male outfield youth soccer players (aged 17.8 ± 0.7 years; height 1.78 ± 0.07 m; body mass 71.1 ± 6.7 kg). All the participants were recruited in a high school three months before testing and executed a familiarisation evaluation one month before the actual tests. The participants were sport students and students in soccer education for five hours per week. The inclusion criteria were that they were part of a team competing in the first division of the regional U18 league and could participate without any injuries on test days.

The players reported an average of one match per week and had been part of a soccer team for 11.7 ± 1.3 years. Moreover, they reported to have practiced soccer in school and club for 9.4 ± 0.4 h per week and participated in self-organised soccer training for 1.3 ± 0.9 h per week. The players agreed to take part in the study, and they signed written consent forms according to regulations of the Norwegian Centre for Research Data. Approval to use the data and to conduct the study was given by the Norwegian Centre for Research Data (reference code nr. 835109).

### 2.2. Design and Methodology

This study developed three tests with the purpose of comparing player performance in ball reception using both technical skills tests and more match-realistic situations such as different SSGs. In the technique test, a ball projection machine (BPM) was used to serve balls with accurate velocity and trajectory to the player, who attempted to direct the ball to a given area. In the first SSG (4vs1), ball receptions were analysed using a 5 × 5 m pitch. In the second SSG (5vs5, including goalkeeper), the pitch size was 32 × 40 m. All player–ball involvements were tracked and recorded using a video camera (Sony PXW-Z90, Sony, Tokyo, Japan). Two educated soccer trainers (UEFA B-license) with more than 10 years of experience as soccer coaches scored every reception of the ball on the basis of set criteria (see Table 1). The criteria were developed as a modification of previously determined [12,22] criteria for scoring soccer skills. All tests were conducted in an indoor soccer hall with artificial turf, and similar balls were used during all tests. The players were tested 3 times, always at in the morning in a period of one month. During test days, the players were interviewed to confirm information regarding their soccer histories During test days, the players were interviewed to confirm information regarding their soccer histories, daily training, match frequency, and levels of match play.

### 2.3. Methods for Testing Ball Reception

Isolated technique test of reception of the ball on the ground and in the air.

**Figure 1:**
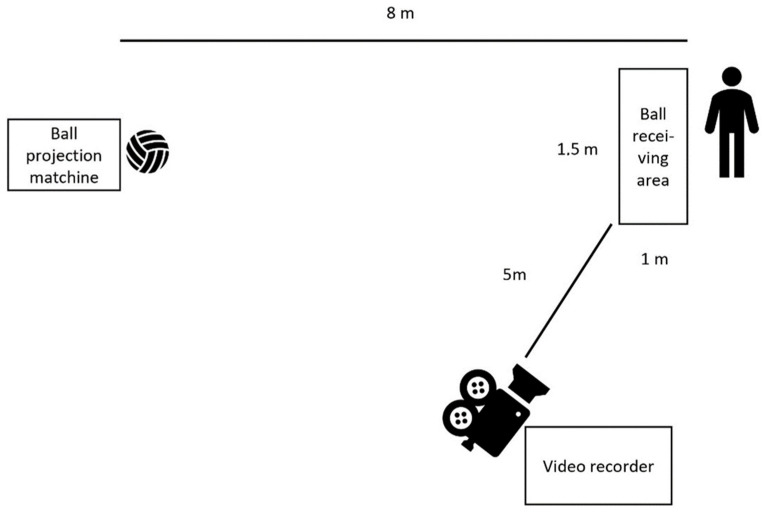
The isolated technique test of receiving the ball from a ball projection machine.

Before testing, the players took part in a standardised general warm-up (15 min), including running with a ball through a curved path of cones, passing and reception 2 and 2 players. An isolated technique test was developed (a modification of an earlier test [25]) to measure the players’ quality of receiving the ball on the ground and in the air. The players stood 8 m from the BPM (Sports Tutor, Burbank, CA, USA), and they were instructed to receive the ball with the purpose of keeping the ball within a marked area in front of them (1 × 1.5 m, see Figure 1). The players were allowed one touch reception followed by one touch to pass the ball to the BPM. The players completed 10 attempts at receiving the ball on the ground with a ball speed of 19 ± 0.5 km/h (measured with a laser gun (Stalker Pro II+, Richardson, TX, USA). They were instructed to receive every second time to the right or left. After a 10 min active break, the players completed 10 attempts at receiving the ball in the air (at a height of approximately 0.2 m) with ball speed at 31 ± 1.2 km/h.

### 2.4. Reception in 4vs1 SSG

Four players (randomly selected) stood on lines in a 5 × 5 m area with one defender inside, chased the ball, and tried to win it. The aim of the four players was to pass the ball without interruption from the defender. For each time the defender won the ball, a new game started immediately with a new ball. The players were instructed to always make two touches, i.e., one touch for reception and one for the pass. The two-touch requirement was to ensure a high number of receptions, although this requirement sometimes felt unnatural for the players in this type of play. The exercise lasted 4 min without breaks other than a change of defender every minute. After a 5 min recovery, the players repeated the same 4vs1 play, but with a change of both teammates and defenders. Every reception of the ball for each player was analysed and given a score related to the quality of the performance (see Table 1), and the total number of receptions for all players was counted in analysing 8 min of SSG.

### 2.5. Reception in 5vs5 SSG

The 5vs5 SSGs were conducted on a pitch size of 32 × 40 m. The games were played according to the official rules of soccer, with the addition of mandatory two-touch ball contact, keeper starting with the ball when the ball goes outside the playing area, and no offside. Each player took part in three teams to ensure that teammates’ quality of skills would have limited influence on performance in receiving the ball [17]. The participants were instructed to execute two touches on the ball every time they received it to ensure a high number of receptions for analysis, although this requirement sometimes felt unnatural to the players. To limit the influence of player position on quality of reception, we organised the teams with two players on defence and two players in attacking positions, and the players changed positions after 3 (out of 6) min. Every reception of the ball by each player was analysed and given a score related to the quality of the performance (see Table 1)**,** and the total number of receptions for all players was tallied during 18 min of SSG.

### 2.6. Statistical Analyses

Data are expressed as mean ± standard deviation (SD) or as individual values. Repeatability analysis was performed on a subset of 40 samples randomly chosen and assessed at two different time points by two raters (coaches). Mean estimates with 95% confidence intervals (CI) were reported for intraclass correlation coefficient (ICC). Interpretation was as follows: <0.50 poor; from 0.50 to 0.75 fair; from 0.75 to 0.90 good; and above 0.90 excellent. The ICC for inter-rater reliability between coaches was from good to excellent at 0.91 (0.78–0.96).

To test for rank order relationship in quality of reception between different reception tests and number of receptions in SSGs, we determined correlations using Spearman’s rank order correlation coefficient (r_s_). The magnitudes of the correlation coefficients were stratified into groups as follows: trivial (r < 0.1), small (0.1 < r < 0.3), moderate (0.3 < r < 0.5), large (0.5 < r < 0.7), very large (0.7 < r < 0.9), nearly perfect (r > 0.9), and perfect (r = 1.0) [26,27]. All statistical analyses were performed using SPSS Statistical Analysis Software for Windows^®^ (SPSS, version 25, Chicago, IL, USA).

## 3. Results

**Figure 2:**
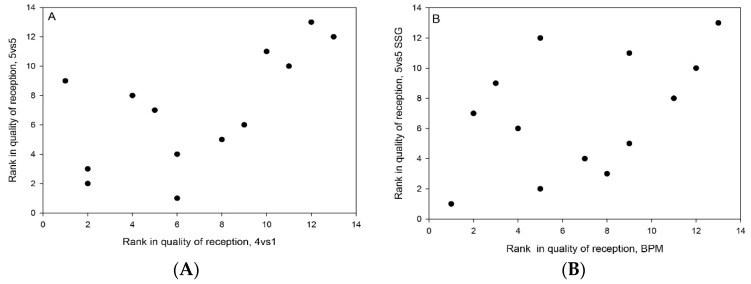
(**A**) Illustration of individual rank in quality of receptions in 4vs1 vs. 5vs5 SSG (r_s_ = 0.61, *p* > 0.05) and (**B**) individual rank in quality of receptions from ball projection machine (BPM) vs. 5vs5 SSG (r_s_ = 0.48, *p* > 0.10).

**Figure 3:**
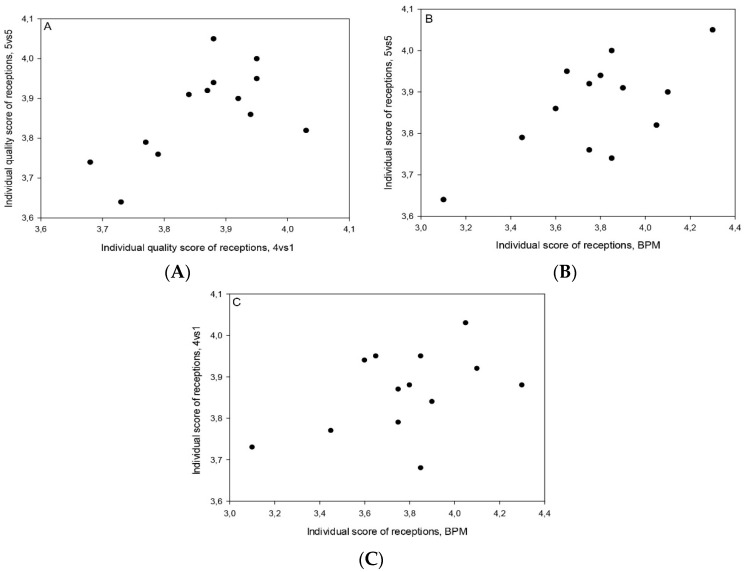
(**A**) Individual plot of quality score in receptions in 4vs1 vs. 5vs5 SSG; (**B**) in ball projection machine (BPM) vs. 5vs5 SSG; and (**C**) in BPM vs. 4vs1 SSG.

A significant correlation was found between 4vs1 SSG and 5vs5 SSG in quality of ball reception (see Table 2 and Figure 2). This study also found a trend towards moderate correlation between quality of ball reception from a ball projection machine and 5vs5 SSG (see Table 2 and Figure 2). No significant correlation was found between quality of ball reception from a ball projection machine and 4vs1 SSG (see Table 2). Individual values of reception quality scores using different reception tests are presented in Figure 3.

**Figure 4:**
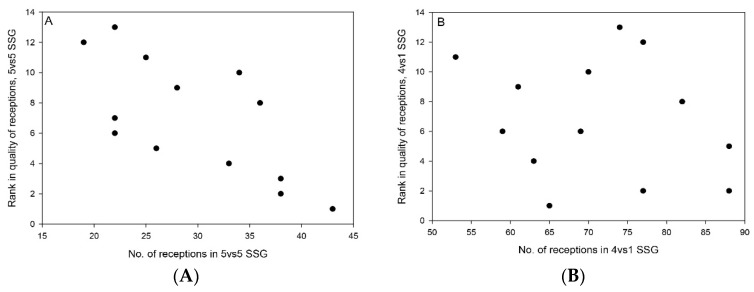
(**A**) Individual plot of rank in quality of receptions in 5vs5 SSG and number of receptions in 18 min of 5vs5 SSG (r_s_ = −0.70, *p* < 0.01); (**B**) plot of rank in 4vs1 SSG and number of receptions in 8 min of 4vs1 SSG (r_s_= −0.18, *p* = 0.56).

A significant correlation was found between number of receptions in 5vs5 SSG and quality score of reception in 5vs5 SSG (r_s_ = −0.70, *p* < 0.01), whereas no correlation was found between number of receptions in 4vs1 SSG and quality score of reception in 4vs1 SSG (r_s_ = −0.18, *p* = 0.56) (see Figure 4). Descriptive statistics (mean ± SD, min–max, CV%) of reception scores were 3.78 ± 0.30, 3.10–4.30, 3.6% for BPM, whereas the respective descriptive statistics were 3.86 ± 0.10, 3.68–4.03, 2.6% and 3.86 ± 0.11, 3.64–4.05, 2.8% for 4vs1 and 5vs5, respectively.

## 4. Discussion

The purpose of this study was to compare soccer players’ skill of ball reception in a soccer technical skills test in relation to reception performance in two different SSG settings.

Our findings demonstrated a significant correlation in quality of receptions between 4vs1 and 5vs5 SSG, as well as a trend towards moderate correlation in quality of ball reception between a technical reception test and 5vs5 SSG. No correlation was found in quality of receptions between 4vs1 and a technical skills test. In addition, this study found a significant correlation between the number and quality of receptions in 5vs5 SSG.

A significant correlation was found in reception performance between the two SSGs. Tactical requirements were higher in 5vs5 compared to 4vs1 SSGs due to higher demands for physical movement, more opponents, and players being required to make or deny goals. In contrast to 4vs1 SSG, physiological factors, especially endurance, can play a decisive role in performance in 5vs5 SSG [24]. However, the players were familiar with the duration and intensity of these 5vs5 SSGs, and the analyses did not find any apparent decrease in reception performance throughout the SSGs. Quality of reception was judged in both SSGs on the basis of both technical and tactical skills, where the players’ actions in relation to match tactics and their technical execution were evaluated.

Our findings of a trend towards a moderate correlation between the technical test using the ball projection machine and reception performance in 5vs5 SSGs indicate that a high level of technical ability may provide players a greater opportunity to succeed in 5vs5 SSGs. SSGs are a popular method of organising training in soccer, both for youth and elite players [24], and performance in SSGs is found to correlate highly with performance in 11vs11 matches [28]. Since reception performance consists of a combination of technical and tactical skills, where both factors are of high importance to match performance, the players need to master both. Receptions in the 5vs5 SSGs were more similar to those in the technical test than were 4vs1 SSGs in relation to distance and speed of passes received.

In 5vs5 SSG, several factors could influence the quality of receptions on the basis of different technical, tactical, and physical factors. Teammates’ ability to execute good passes and their tactical knowledge of correct positions in relation to the game will influence players’ quality of receptions. When a teammate moves to a good position and is ready to receive a pass, the judgement is simplified for the player regarding direction of reception. In addition, opponents’ quality of defence will influence degree of difficulty in making high-quality receptions.

Our finding of no significant correlation between an isolated technique test and performance in the 4vs1 SSG is consistent with findings regarding preferred methods of measurement for soccer skills [1]. Low validity of technical skill scores in match performance was also found in two other studies [11,12]. However, studies have shown that performance of technical skills can predict performance of dribbling and passing in SSG and match play [13,14,15,16,17]. The ball projection test measured the players’ technical reception skills. In this test, there were no tactical aspects involved as the only task was to control the ball in a 1 × 1.5 m area. In the 4vs1 SSG test, the performance score depended on receiving the ball in both the correct direction on the basis of the opponent’s movement and the correct distance away from the player. During testing, it was observed that having to execute two-touch on the ball was in some instances unnatural for the participants. These situations could have influenced the results regarding reception in 4vs1 SSGs. Furthermore, the passes for reception in 4vs1 were from a short distance and mostly on the ground, making the degree of difficulty for the reception relatively low as pressure from the opposing player was low, while reception from the BPM, especially at the highest ball speed of 31 km/h, presented a high degree of difficulty for the participants.

Quality of reception in these SSGs depends on players’ technical as well as tactical skills. As was the case for the technical skills presented in this study, it is obvious that tactical skills will also vary. The homogeneity of participants in the present study was relatively wide, with players who had participated in soccer clubs for about 12 years and trained in soccer for an average of approximately 10 h per week. Theoretically, players with high levels of technical skills could have low tactical skills, and vice versa. In contrast to our findings of no correlation between technical tests and 4vs1 SSG, a study of young players in an elite Brazilian soccer academy found that general passing ability, measured with a technical passing test, was a good predictor for high passing success in a 3vs1 possession game [15]. In addition, a study [17] found that dribbling speed in an isolated test can be used to predict the possibility of scoring in a one-on-one soccer game. It could be argued that, especially in one study [17], the similarity between the two tests has been found to be high. On the basis of the principle of specificity, high similarity results in a greater chance of finding a high degree of validity in a technical test and performance in SSGs [29].

There are several factors that could influence the performance of a soccer player during SSGs, for example, the effect of the player’s level of technical and tactical skills in terms of the intensity of the game and the performance level of teammates and opponents [18,19]. The intensity in 4vs1 SSG differs from the intensity in the ball projection test. In 4vs1 SSG, the players had to move along the 5-m line between the cones to be in good passing position in relation to their teammates. When players received the ball, they had to spend as little time as possible to move it in the correct direction before passing in order to limit the possibility of the defender winning the ball. The area used in 4vs1 was quite small (5 × 5 m); the duration of the games was only 4 min, and the participants were instructed to only move along the straight line between the cones. However, participants’ tactical skills of being in the right position at the right time, as well as their teammates’ ability to execute good passes, might have affected scores in 4vs1 SSG. Teammates’ offensive skills and the performance level of the opponent will influence quality of reception [21]. However, in the present experiment, participants acted as both teammates and opponents during testing in order to limit these effects. Still, when evaluating technical skills, researchers must be aware of instability in performance [19].

Our results showed a significant correlation in 5vs5 SSG between number of ball receptions and players’ reception scores. A possible explanation for this could be that players who make more receptions in training and matches develop their skill levels to a greater degree than do players who are less involved. This highlights the importance of increased activity and many ball contacts in daily training. No correlation between number of receptions and reception scores was found in 4vs1 SSG. This was probably due to the difference in activity between the two SSGs. In 5vs5 SSG, a player’s involvement and ability to be in the correct position to receive a pass are decisive in executing a high number of passes, while in 4vs1 SSG, where the players had to move only along a 5-m line, low involvement nevertheless resulted in a high number of passes from their teammates.

This study did have some limitations, most notably as only a small sample of 13 soccer players were involved. The group of participants in this study were relatively similar in relation to age and performance level. However, as in most soccer groups, some differences in skills were measured and observed. In the random selection of teams for the SSGs, chance coincidences occurred, according to which teammates and opposing players they were tested together with could have a decided influence on the results. Similar challenges will always be encountered when analysing game activities in soccer, but measures to reduce the degree of bias were implemented. Similarly, increasing the number of matches every player took part in could have strengthened the results. However, the total number of matches contained a reasonable number of receptions to analyse for each player.

## 5. Conclusions

This study aimed to explore the correlation between performance of ball reception scoring in a soccer technical skills test and reception performance in 4vs1 and 5vs5 SSGs, as well as the correlation of quality of reception scores in 4vs1 versus 5vs5 SSGs. Not surprisingly, we found a highly significant correlation in quality of reception between the two SSGs among players who had both technical and tactical skill elements. The trend towards moderate correlations between 5vs5 SSGs and the isolated technical reception test could imply the importance of practicing the technical aspects of soccer. Moreover, it seems like the players with the best reception performance are those players who are most involved in SSGs, that is, they performed the most receptions during SSGs.

## 6. Practical Applications

A vital question for soccer coaches worldwide is how to organise training so that players can enhance their skill levels as much as possible. Undoubtedly, real-game situations are the most specific way to practice the technical and tactical aspects of reception performance. However, for variation purposes and periodisation of training load, it might be recommended that coaches focus on developing player technique both through isolated technique exercises and more match-realistic play such as SSGs.

## Figures and Tables

**Table 1:** Criteria for scoring ball reception from the BPM at speeds of 19 km/h and 31 km/h, 4vs1 SSGs and 5vs5 SSGs, with full-size goal and keepers.

Score	Criteria: Ball Reception, Technical Skill 19 km/t	Criteria: Ball Reception, Technical Skill 31 km/t	Criteria: Ball Reception, Technical and Tactical Skill, 4vs1	Criteria: Ball Reception, Technical and Tactical Skill, 5vs5
1	The player does not manage to stop the ball.	The player does not manage to stop the ball.	The player loses control of the ball from an easy pass of does not manage to stop the ball.	The player loses control of the ball from an easy pass of does not manage to stop the ball.
2	The player loses control over the ball, and the ball goes outside the area in front of or beside the player (1 × 1.5 m) before he manages to pass the ball	The player does not manage to control the ball inside the area, and the ball goes more than 2 m outside the area in front of or beside the player (1 × 1.5 m) before the pass is made	The player loses control of the ball from a difficult pass.	The player loses control of the ball from a difficult pass.
3	The player manages to control the ball in the area, but the ball is not controlled in the correct direction (left or right).	The player does not manage to control the ball in the area, and the ball goes less than 2 m outside the area before the pass is made	The player controls the ball, but the receiving makes it difficult to make a pass in the correct direction.	The player controls the ball, but the receiving makes it difficult to make a pass in correct direction.
4	The player manages to control the ball in the area, in the correct direction (left or right), but the ball is too close or too far from the player, resulting in difficulty in making the pass	The player manages to control the ball inside the area, but the ball is too close or too far from the player, resulting in difficulty in making the pass	The player controls the ball, and the receiving makes it easy to perform a pass in the correct direction.	The player controls the ball, and the receiving makes it easy to perform a pass in the correct direction.
5	The player manages to control the ball within the area, in the correct direction (left or right), and the receiving is perfect, so the pass is easy to perform.	The player manages to control the ball inside the area, and the receiving is perfect for executing the pass.	The player controls the ball in a perfect way, in a difficult situation, with high pressure from the defender.	The player controls the ball in a perfect way, passing an opponent, making progress in the soccer game or managing to control a difficult pass.

**Table 2:** Spearman’s correlation (r_s_) between tests of reception from a ball projection machine (BPM), 4vs1 SSG, and 5vs5 SSG for 13 youth soccer players.

	Rank BPM	Rank 4vs1	Rank 5vs5
Rank BPM	1.000	0.399	0.479
Rank 4vs1	0.399	1.000	0.606 *
Rank 5vs5	0.479 ^#^	0.606 *	1.000

* Correlation is significant at the 0.05 level (two-tailed); ^#^ trend towards correlation (*p* < 0.10, two-tailed).

## Data Availability

Data sharing is not applicable to this article.
